# A Rare Case of Pediatric Nasopharyngeal Rhabdomyosarcoma With Parameningeal Extension Presenting as a Thornwaldt Cyst

**DOI:** 10.7759/cureus.49224

**Published:** 2023-11-22

**Authors:** Rao Adeel, Kaneez Fatima, Muneeb Shahid, Haris Memon, Abul Haque

**Affiliations:** 1 Emergency Department, Broomfield Hospital, Mid and South Essex NHS Foundation Trust, Chelmsford, GBR; 2 Internal Medicine, Nishtar Medical University, Multan, PAK; 3 Radiology, Broomfield Hospital, Mid and South Essex NHS Foundation Trust, Chelmsford, GBR

**Keywords:** thornwaldt cyst, parameningeal disease, parameningeal rhabdomyosarcoma, nasopharyngeal cancer (npc), nasopharyngeal soft tissue mass, nasopharyngeal rhabdomyosarcoma

## Abstract

Rhabdomyosarcoma (RMS) is a common soft tissue malignancy of the pediatric age group, frequently involving the head and neck region; however, nasopharyngeal RMS is a rare entity. By virtue of its parameningeal involvement and nonspecific presentations, nasopharyngeal RMS has become a clinical challenge to diagnose. We present a case of an eight-year-old boy presenting with signs and symptoms of nasal obstruction who was initially being treated for tonsillitis. Recurrent episodes led to detailed examination and radiologic imaging, and a diagnosis of Thornwaldt cyst was made. For the relief of symptoms, a debulking surgery was performed followed by a biopsy which revealed the mass to be a nasopharyngeal RMS. Our case highlights the importance of including nasopharyngeal RMS into the differentials of midline nasal masses along with the role of biopsy for confirming its diagnosis as treatment modalities for it are strikingly different than the other more common group of conditions, i.e., benign nasal masses. While surgery is usually delayed in the latter group, it can be of prime importance while treating nasal RMS, along with chemotherapy and radiotherapy.

## Introduction

Rhabdomyosarcoma (RMS) is a highly malignant pediatric soft tissue tumor of the skeletal muscles originating from primitive mesenchymal cells. Despite being the most common soft tissue malignancy in children, RMS is still rare as it accounts for only 3% of all childhood tumors [1]. Although RMS can be present anywhere in the body, they have a predilection for the head and neck area involving the orbital, non-parameningeal, and parameningeal sites. Amongst all, nasal RMS is rare, as it involves only 10-15% of all head and neck RMSs [2].

Regarding clinical features, head and neck RMS usually manifest with vague symptoms of nasal obstruction, epistaxis, recurrent otitis media, tonsillitis, and proptosis [3]. They also show a high preponderance of orbital region involvement which allows them to get diagnosed earlier as compared to those involving the parameningeal sites because of their invisible clinical appearance [4]. As a result, parameningeal RMS is diagnosed at an advanced stage and shows more aggressive behavior and a poorer prognosis.

As established by the Intergroup Rhabdomyosarcoma Study Group (IRSG), the ideal treatment for RMS is multimodal and includes a combination of surgery, chemotherapy, and radiotherapy [5]. Therefore, early diagnosis and ideal management of this rare tumor can improve survival chances.

## Case presentation

An eight-year-old boy presented in the emergency department with chief complaints of shortness of breath. He had been taken to his local doctor a couple of weeks back where a diagnosis of recurrent tonsillitis was made and antibiotics were prescribed, but they all proved unhelpful. His symptoms had worsened progressively and significantly over the past three weeks, including snoring, mouth breathing, difficulty eating and drinking, as well as headaches. He had also lost some weight and stopped attending school. 

On physical examination, the young boy appeared alert and afebrile. Despite shortness of breath and mouth breathing, he was able to speak in full sentences but with a nasal twang. His oral examination revealed swollen tonsils of grade 3 without exudate. The rest of the systemic examination was unremarkable. During his admission, he was observed overnight, and apneas and desaturations were noted during sleep.

**Figure 1:**
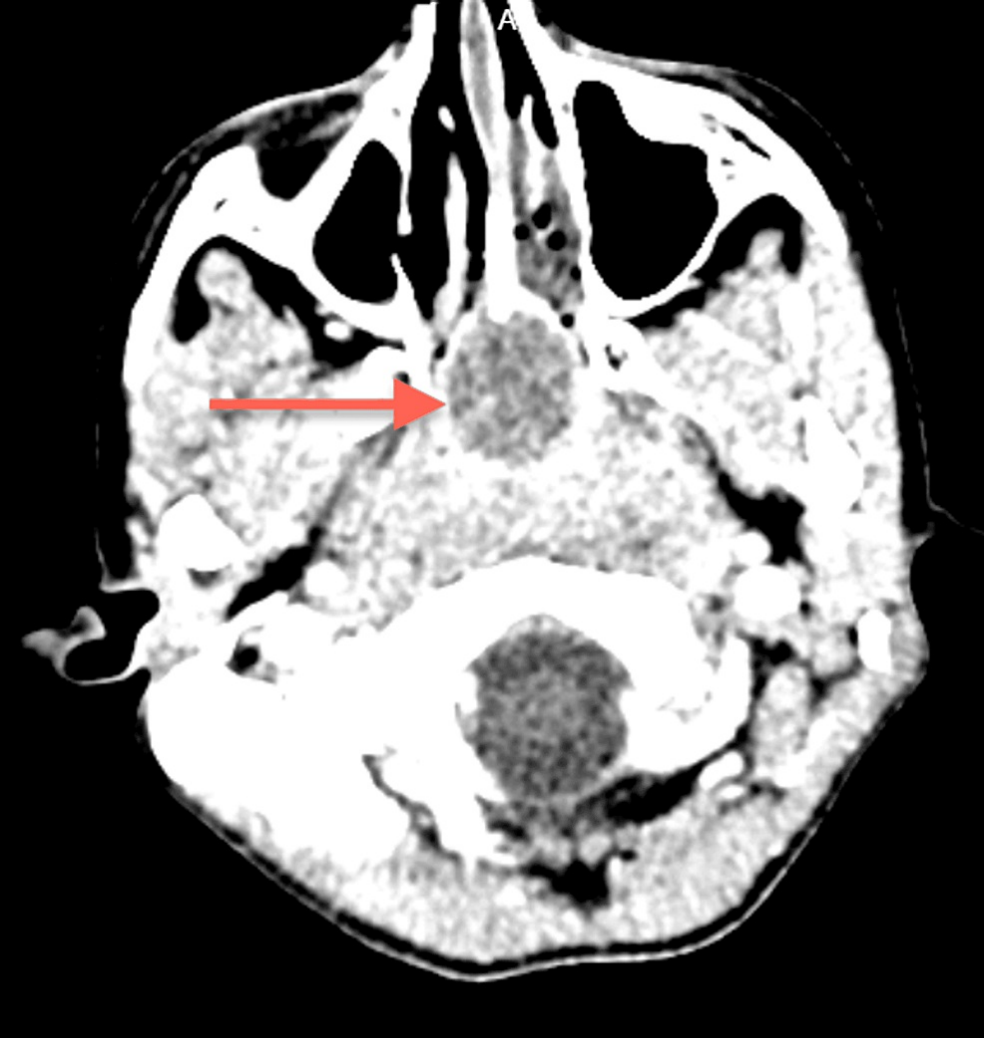
CT Neck CT of the neck showing the adenoid region with a 4.6x2.7 mm cystic lesion remodelling the floor of the sphenoid sinus, displacing the uvula anteriorly and compromising the nasopharyngeal airspace.

**Figure 2:**
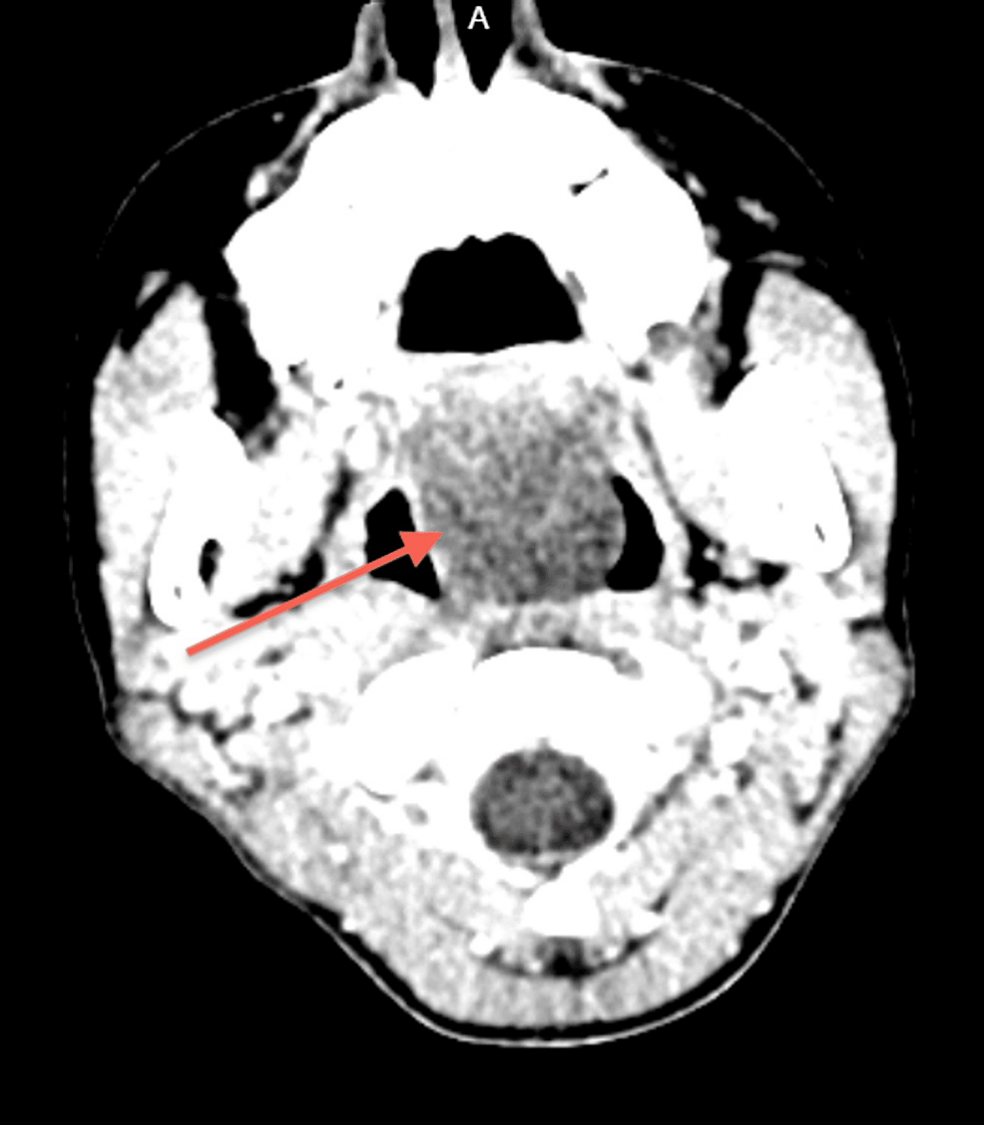
CT Neck

**Figure 3:**
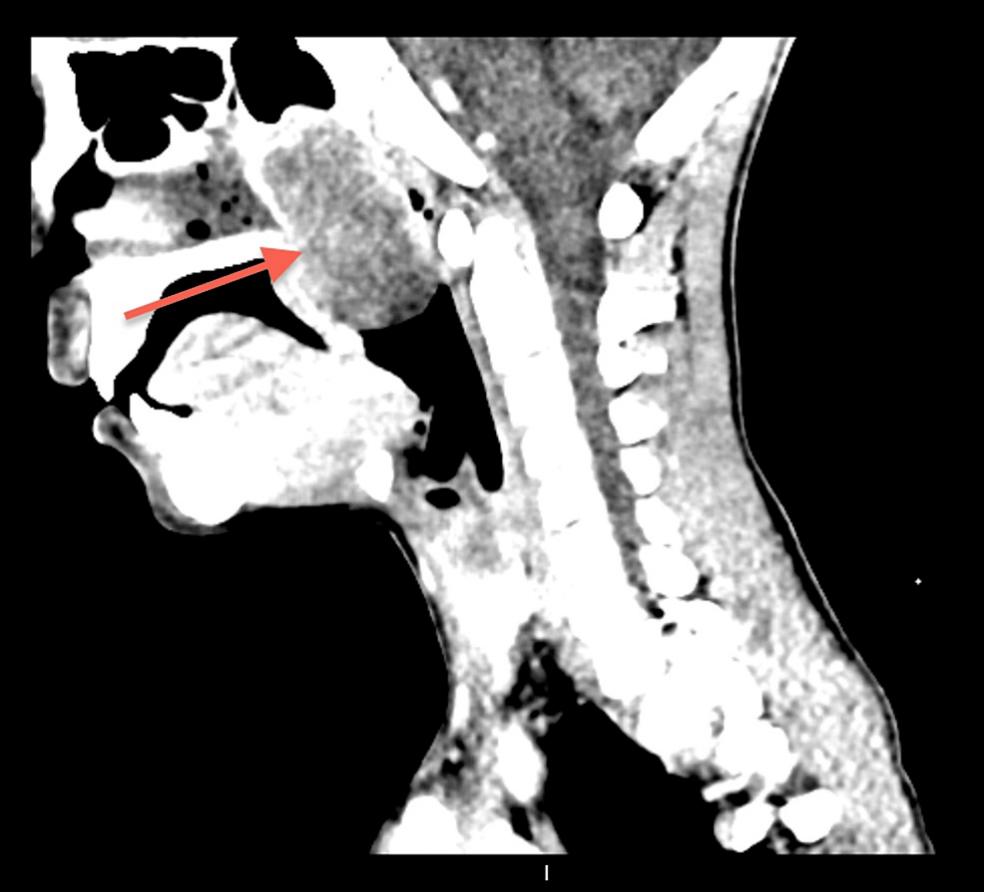
CT Neck Sagittal View CT of the neck showing the adenoid region with a 4.6x2.7 mm cystic lesion remodeling the floor of the sphenoid sinus, displacing the uvula anteriorly and compromising the nasopharyngeal airspace.

**Figure 4:**
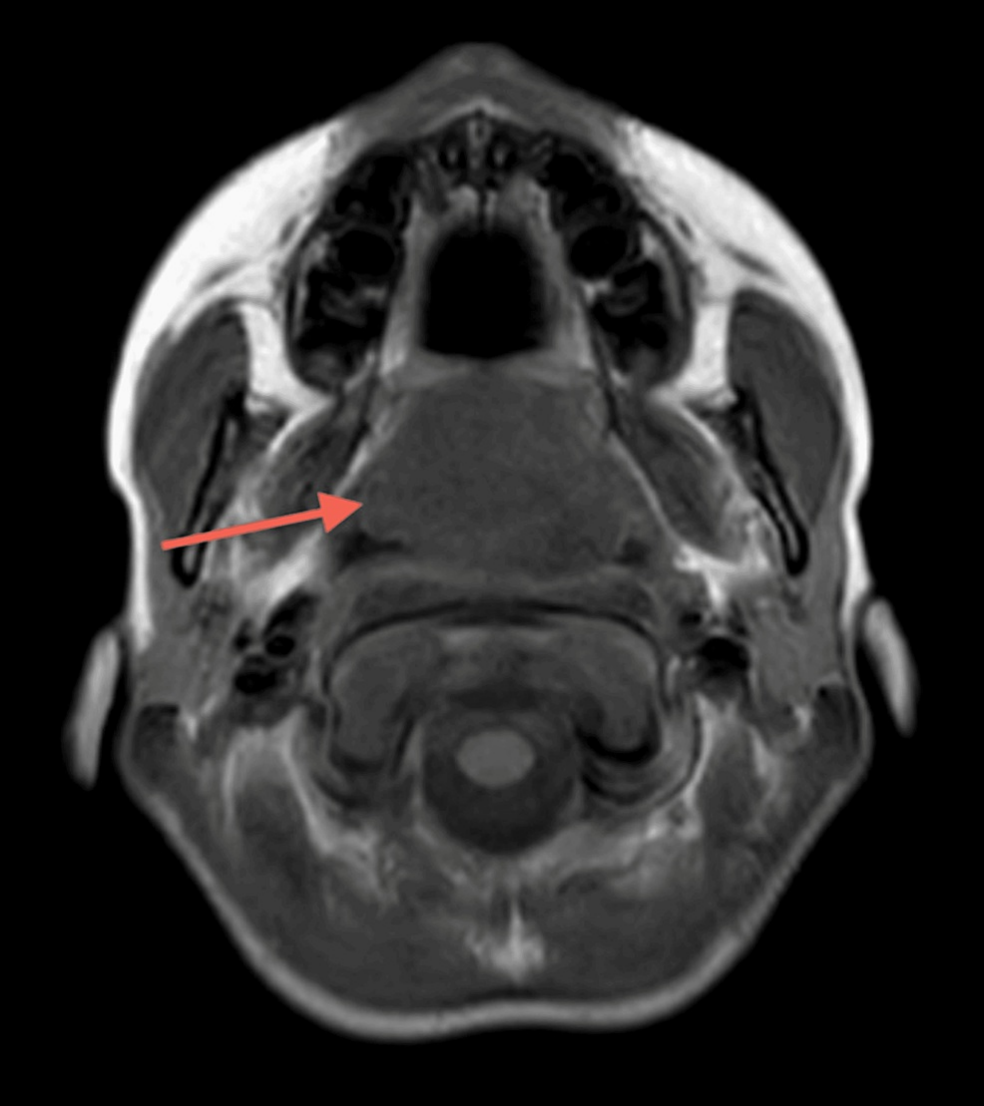
MRI Neck (T1) The large nasopharyngeal well-circumscribed cyst measures 47 x 28 x 35 mm. The sheer volume of this Thornwaldt cyst is remodeling the undersurface of the sphenoid sinus. The cyst is anteriorly eroding the posterior edge of the hard palate and midline vomer segment of the nasal septum, the uvula is displaced significantly into the oral cavity. Parapharyngeal recesses are preserved. No intracranial extension.

**Figure 5:**
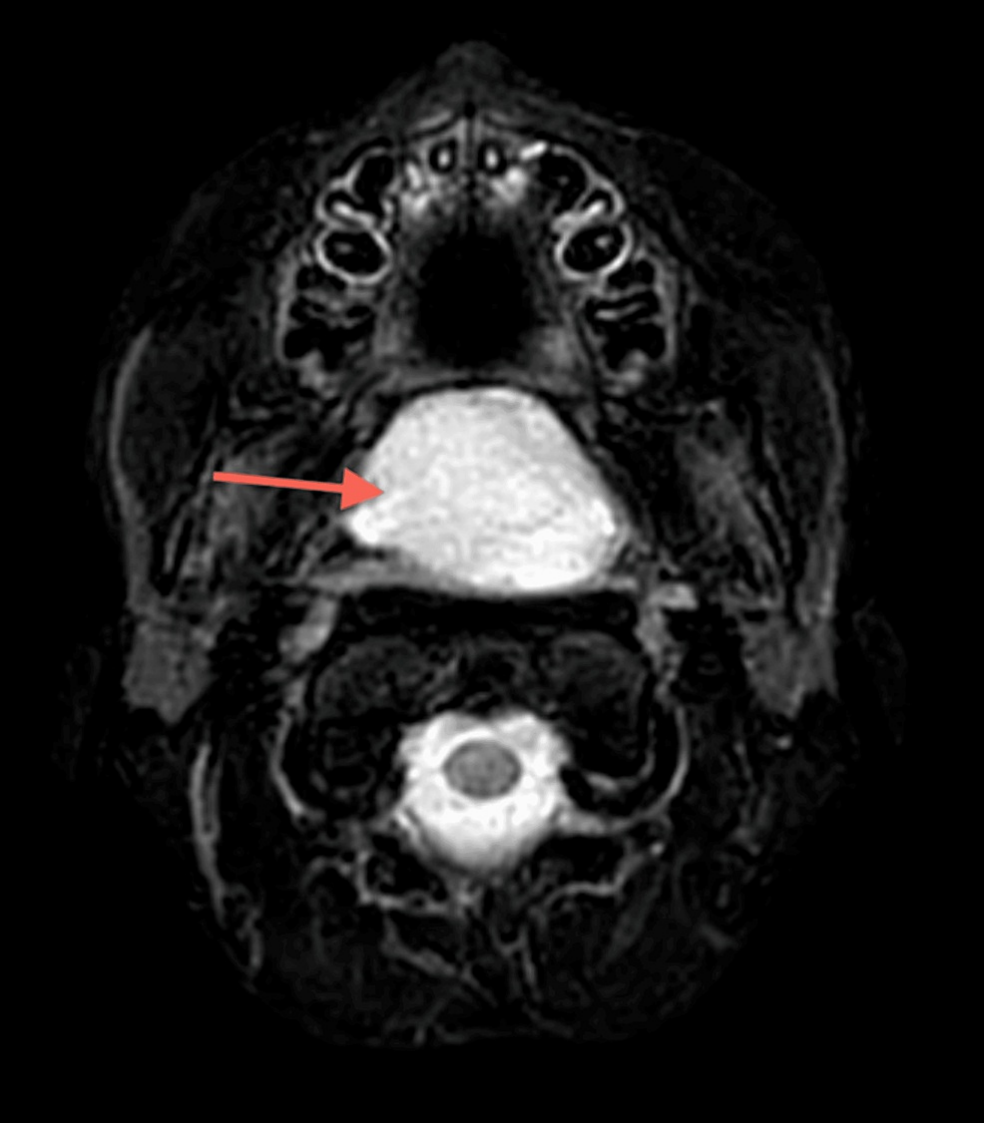
MRI Neck (T2) The large nasopharyngeal well-circumscribed cyst measures 47 x 28 x 35 mm. The sheer volume of this Thornwaldt cyst is remodeling the undersurface of the sphenoid sinus. The cyst is anteriorly eroding the posterior edge of the hard palate and midline vomer segment of the nasal septum, the uvula is displaced significantly into the oral cavity. Parapharyngeal recesses are preserved. No intracranial extension.

**Figure 6:**
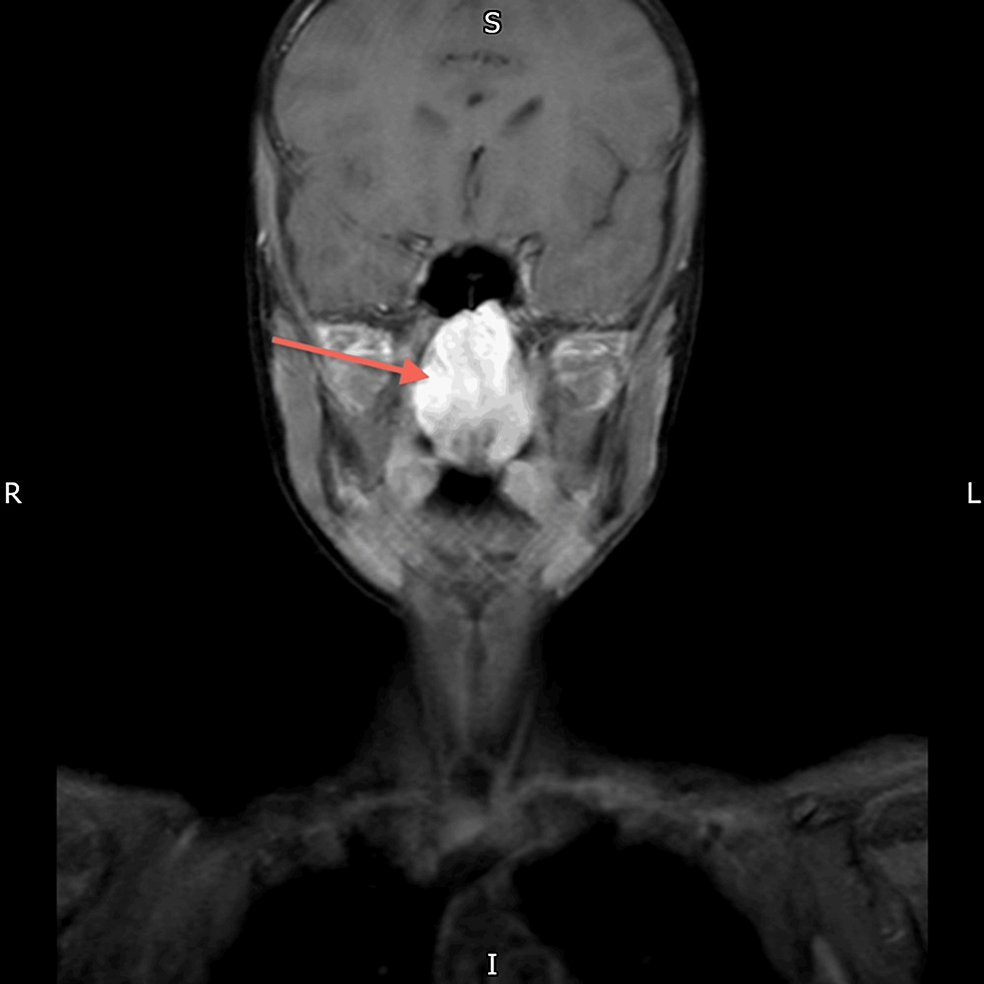
MRI Neck (Post Contrast) The large nasopharyngeal well-circumscribed cyst measures 47 x 28 x 35 mm. The sheer volume of this Thornwaldt cyst is remodelling the under surface of the sphenoid sinus. The cyst is anteriorly eroding the posterior edge of the hard palate and midline vomer segment of the nasal septum, the uvula is displaced significantly into the oral cavity. Parapharyngeal recesses are preserved. No intracranial extension.

**Figure 7:**
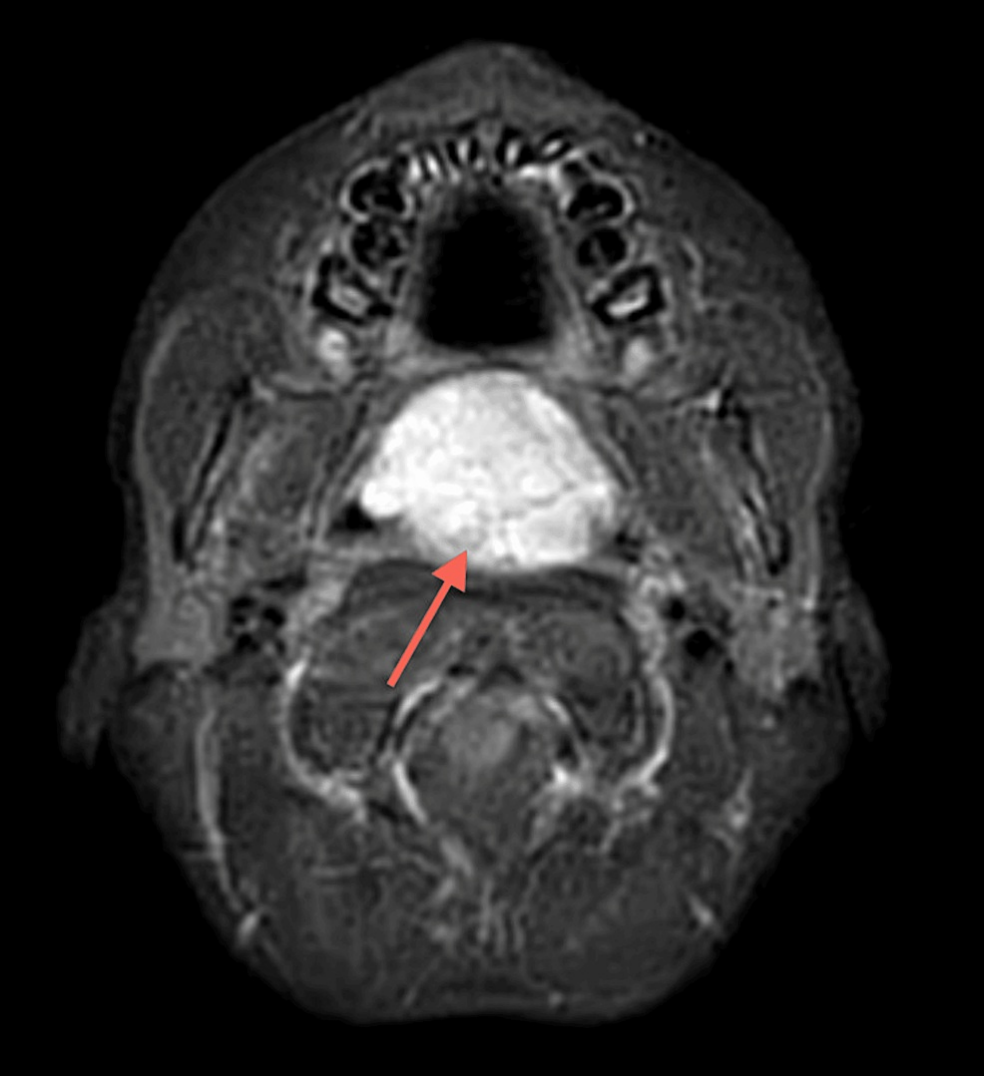
MRI Neck (Post Contrast) The large nasopharyngeal well-circumscribed cyst measures 47 x 28 x 35 mm. The sheer volume of this Thornwaldt cyst is remodeling the undersurface of the sphenoid sinus. The cyst is anteriorly eroding the posterior edge of the hard palate and midline vomer segment of the nasal septum, the uvula is displaced significantly into the oral cavity. Parapharyngeal recesses are preserved. No intracranial extension.

**Figure 8:**
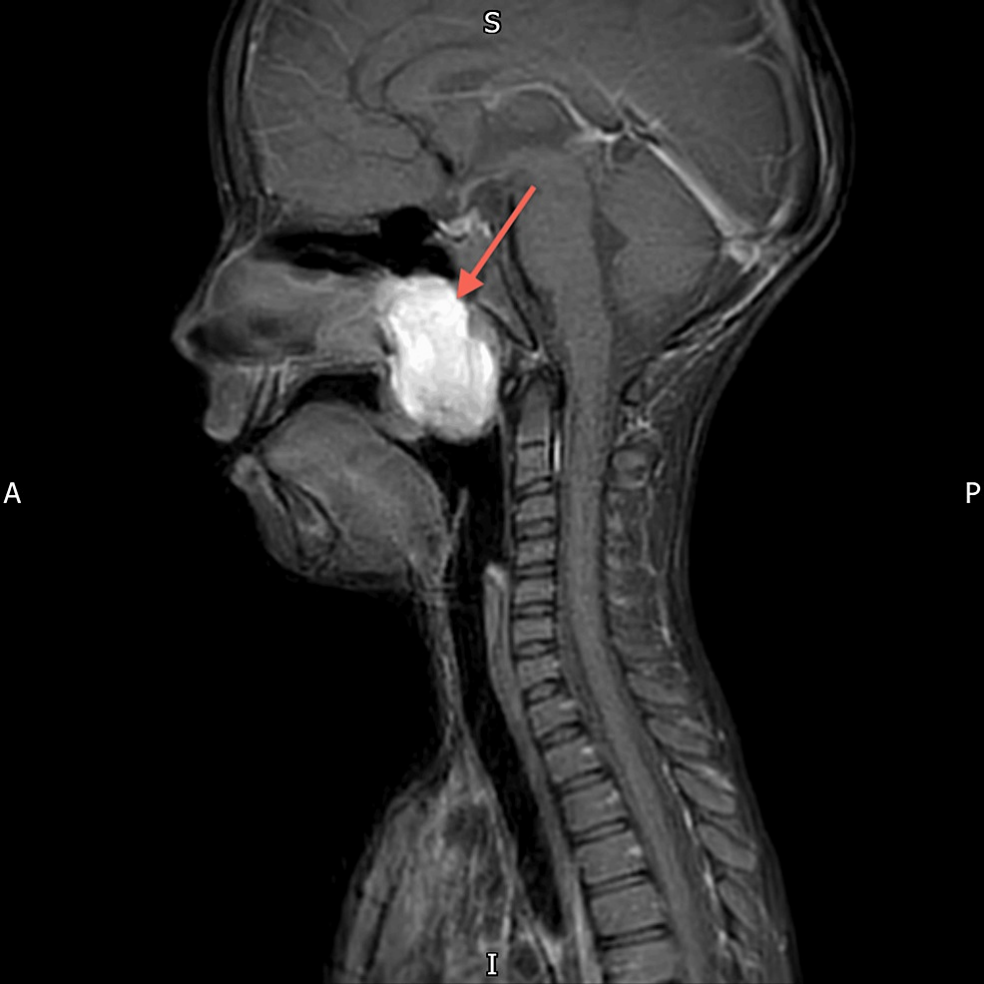
MRI Neck (Post Contrast) The large nasopharyngeal well-circumscribed cyst measures 47 x 28 x 35 mm. The sheer volume of this Thornwaldt cyst is remodeling the undersurface of the sphenoid sinus. The cyst is anteriorly eroding the posterior edge of the hard palate and midline vomer segment of the nasal septum, the uvula is displaced significantly into the oral cavity. Parapharyngeal recesses are preserved. No intracranial extension.

The ENT department was consulted, and a probable diagnosis of retro nasal/pharyngeal abscess was made. We undertook a CT scan of the head and neck (Figures 1-3). CT scan revealed a cystic lesion of 4.6x2.7 cm in the adenoid region that appeared to be a Thornwaldt cyst, compromising the nasopharyngeal airway space. Following that, a head and neck MRI was carried out, affirming the previous findings and adding that the cyst is appreciably remodeling the nasopharyngeal boundaries (Figures 4-8).

To relieve the symptoms, a debulking surgery was done. A biopsy was taken and sent for histopathological examination. A diagnosis of rhabdomyosarcoma was confirmed. Following the RMS 2005 Guidelines, a high-risk group treatment protocol was started and sessions for vincristine chemotherapy were scheduled. The treatment response has been good so far. 

## Discussion

The second most common cause of death in pediatric patients following trauma is pediatric malignancies. Out of these malignancies, head and neck tumors are relatively a rare group accounting for up to 12% of the total. The incidence of head and neck tumors is 1.49 cases per 1,000,000 patient-years. Patient-years represent the number of patients multiplied by the number of years of observation [6]. Rare incidences are one factor in the limited clinical understanding of pediatric RMS. Furthermore, the constantly changing and evolving guidelines regarding the diagnosis and treatment of pediatric RMS pose another challenge for its study. However, its rare occurrences and the aggressive nature both emphasize the importance of continued and improved study regarding this tumor so that agreed guidelines and a thorough understanding can be made which will guide rationale-based treatment for improved patient benefits ​[4]. 

A major challenge hindering its early diagnosis is the non-specific nature and diversity of its symptoms, which overlap with so many common and benign conditions that the diagnosis is usually made only in the advanced stage. One study was based on recording symptoms in patients of pediatric RMS. Nine cases presented with nasal obstruction as the chief complaint while another three presented as cases of otitis media ​[5].​ 

Another study revealed that sarcomas of the sino-nasal region presented with symptoms of nasal obstruction, epistaxis, and sinus pain which overlapped with many other benign conditions like sino-nasal polyposis, allergic rhinitis, or chronic sinusitis leading to difficulty in their early diagnosis. This is attributed to their location in the sinuses or nasal cavity [7]. 

Our reported case was diagnosed earlier as a Thornwaldt cyst, which is a developmental benign midline lesion presenting in the nasopharynx. Some studies revealed its incidence to be around 1.4-3.3 %. Most cysts are undiagnosed because of their small size; however, some may grow larger and present with symptoms such as headache, nasal obstruction, postnasal drip, or dysfunction of the Eustachian tube [8].

In a case reported by Larson et al., a nine-month-old child presenting with a 1 cm firm, slightly mobile mass over the nasal dorsum was suspected to be either nasal dermoid or neuroglial heterotopia considering MRI findings. Surgical excision was carried out due to intracranial extension; however, biopsy findings confirmed it to be embryonal RMS [9]. 

A similar case study mentions a healthy four-year-old child presenting with difficulty to eat, odynophagia and neck pain who was later diagnosed to have peritonsillar abscess peritonsillar abscess (PTA) based on examination findings of fullness in the right peritonsillar arch and anterior lymphadenopathy on his subsequent visits. These findings were also supported by a CT scan. He was taken to the OT; however, a nasopharyngeal mass was found instead of PTA and a subsequent biopsy confirmed it to be embryonal RMS [10]. 

To the best of our knowledge, the current case is the only reported case of pediatric nasopharyngeal RMS diagnosed and operated as Thornwaldt cyst. This emphasizes the importance of including nasopharyngeal RMS in the differentials of a nasopharyngeal mass as it can affect the treatment modality emphasizing the need for early surgery which is usually avoided in cases of other benign lesions due to the developing nature of surrounding structures. The present case report also emphasizes the need for a biopsy performed on midline nasal masses as radiological investigations can lack specificity in making the diagnosis of nasopharyngeal RMS.

## Conclusions

The case report highlights the importance of including nasopharyngeal RMS in the differentials of midline nasal masses. Understanding the broad clinical spectrum of this condition and the importance of biopsy findings for its diagnosis are also highlighted. Late diagnosis due to incomplete understanding of the varied presentations and appropriate investigations can lead to poor prognosis.
